# Pleiotropic Effects of Metformin on Cancer

**DOI:** 10.3390/ijms19102850

**Published:** 2018-09-20

**Authors:** Hans-Juergen Schulten

**Affiliations:** Center of Excellence in Genomic Medicine Research, King Abdulaziz University, P.O. Box 80216, Jeddah 21589, Saudi Arabia; hschulten@kau.edu.sa; Tel.: +966-12-640-1000-25482

**Keywords:** Metformin, cancer, pleiotropic effects, scientific studies, population-based studies, clinical trials

## Abstract

Metformin (MTF) is a natural compound derived from the legume *Galega officinalis*. It is the first line antidiabetic drug for type 2 diabetes (T2D) treatment. One of its main antidiabetic effects results from the reduction of hepatic glucose release. First scientific evidence for the anticancer effects of MTF was found in animal research, published in 2001, and some years later a retrospective observational study provided evidence that linked MTF to reduced cancer risk in T2D patients. Its pleiotropic anticancer effects were studied in numerous in vitro and in vivo studies at the molecular and cellular level. Although the majority of these studies demonstrated that MTF is associated with certain anticancer properties, clinical studies and trials provided a mixed view on its beneficial anticancer effects. This review emphasizes the pleiotropic effects of MTF and recent progress made in MTF applications in basic, preclinical, and clinical cancer research.

## 1. Introduction

Metformin (MTF) is a 1,1-dimethylbiguanide that is derived from the legume *Galega officinalis* and was first reported as an antidiabetic drug in 1957 [1]. MTF is a hydrophilic compound that is mostly present as a positively charged species under physiological conditions. Based on its safety profile, MTF is the first-line antihyperglycemic drug for treatment of type 2 diabetes (T2D) which represents more than 90% of diagnosed diabetes [2,3]. T2D is characterized by insulin resistance in liver, muscle, adipose tissue, and other insulin-resistant tissues which leads to hyperglycemia and secondary hyperinsulinemia [4]. The main effect of MTF in diabetic patients is to lower glucose levels, resulting in secondary reduction of insulin levels. One of the main effects of MTF in the liver is lowering of gluconeogenesis and reduction of hepatic glucose release [5,6,7].

In contrast to other antidiabetic pharmaceuticals, MTF exhibits only rare side effects such as hypoglycemia, hyperinsulinemia, vitamin B12 deficiency, peripheral neuropathy, or very rarely, lactic acidosis and is less associated with diabetic-related risk factors in overweight patients [8,9,10,11]. Insulin is seemingly associated with growth promoting effects under certain conditions [12]. The U.S. Food and Drug Administration (FDA) approved MTF in 1994 for T2D treatment. 

Scientific evidence that linked MTF to reduced cancer risk came from studies in high fat-fed hamsters where a pancreatic carcinogen failed, in MTF-treated animals, to induce pancreatic cancer derived from the islets [13]. In contrast, non-MTF-treated hamsters revealed cancerous lesions and, in addition, had significantly more hyperplastic and premalignant lesions than the MTF-treated group. Epidemiological studies indicated that obesity and T2D, but not T1D, are associated with elevated relative risk for certain cancer types including liver, biliary tract, pancreatic, colorectal, kidney, bladder, breast, and endometrial cancer [3,14]. Common confounding factors shared between diabetic and cancer patients are obesity and low physical activity.

MTF exerts its primary main effects at the molecular level as an oxidative phosphorylation (OXPHOS) inhibitor by reversibly inhibiting NADH dehydrogenase (mitochondrial complex I) activity of the respiratory chain, resulting in suppression of ATP production [15,16,17]. The AMP-activated protein kinase (AMPK) is a key molecule by which MTF exerts a substantial part of its pleiotropic effects [18]. The whole spectrum of MTF anticancer effects on the molecular and cellular levels is subject of several in vitro and in vivo studies. Observational studies, clinical trials, and meta-analyses are undertaken to assess beneficial effects of MTF on cancer treatment. This review aims to provide a comprehensive overview of the current knowledge of MTF applications in cancer research with an emphasis on the underlying molecular biology effects of MTF. 

## 2. MTF Bioavailability 

Cellular uptake and expulsion rates for MTF depend largely on the expression of organic cation transporters (OCT1, OCT2, and OCT3) and on multidrug and toxin extrusion proteins (MATE1 and MATE2) [19]. MTF is transported into enterocytes at the apical membrane by plasma membrane monoamine transporter (PMAT; alias, SLC29A4) and OCT3 while it is transported out of the enterocytes at the basolateral membrane by OCT1 [20]. OCT1 and OCT3 are the hepatocyte influx transporters for MTF while MATE1 is the hepatocyte efflux transporter. MTF is transported from the circulation into renal epithelial cells by OCT2 and transported into urine by MATE1 and MATE2. Orally administered MTF is primarily taken up through the upper small intestine where it accumulates beyond its plasma concentrations. Of note, double knockout mice for both Oct1 and Oct2 transporters revealed a significant reduction in MTF clearance and distribution but mostly did not affect tissue distribution or pharmacodynamics of MTF [21].

The bioavailability of orally administered MTF is 40–60% [22]. A 1.5 g MTF dose achieves after 3 h a peak plasma concentration of ~18 µM (~3 mg/L) [6]. MTF uptake is dose-dependent, but saturable [23]. Plasma levels did not exceed ~30 µM (~5 mg/L) in clinical trials. The plasma elimination half-life is about 5–6 h in patients with normal kidney function and who received multiple MTF applications [24]. About 90% of orally administered MTF is excreted via the kidneys within 24 h. An exploratory study in pancreatic patients enrolled in a phase II study found that those who received gemcitabine, erlotinib, and MTF (2 g daily) and achieved MTF plasma concentrations >1 mg/L were more likely to have an overall survival benefit than those with a lower MTF concentration; however, higher MTF concentrations were less likely to occur in patients with advanced cancer and gastrointestinal implications who had poor prognosis [25].

The half-maximal inhibitory concentration (IC_50_) of MTF ranged between 5 and 20 mM in cell line models but differed under certain conditions. In breast cancer cell lines, IC_50_ of MTF increased under high glucose levels and MTF-induced apoptosis and cell cycle arrest were less pronounced under high than under low glucose levels [26]. An MTF concentration of 1 mM is required to induce AMPK activation in cell culture [27]. This corresponds to an intracellular concentration of 131 µM, which is similar to a 145 µM plasma concentration in mice receiving intraperitoneal (i.p.) injected MTF. 

Compared to oral MTF gavage, i.p. administration resulted in higher MTF bioavailability in mice; in particular, it led to pronounced higher MTF concentrations in blood, liver and kidneys, and stimulated retention of the glucose analog fluorodeoxyglucose (FDG) in kidneys [28]. In tumor mice models, biodistribution of intravenous (i.v.)-applied ^11^C-MTF revealed comparably high tracer uptake in the kidneys and liver, whereas tracer uptake in blood and tumors was comparably low. The authors suggested that ^11^C -MTF-positron emission tomography (PET), possibly in combination with mutational screening of genes related to MTF sensitivity, could become clinically practicable to identify MTF-sensitive tumors. In a rat model of lipopolysaccharide-induced systemic inflammation, MTF concentrations varied between different brain regions with the highest MTF concentrations measured in the pituitary gland and cerebrospinal fluid (~30 μM) [29]. A critical review stated that the considerable effects of MTF demonstrated in various experimental studies are partly attributed to the applied supra-pharmacological MTF concentrations that are unlikely to be achieved in patients [20].

## 3. Pleiotropic MTF Effects on Cancer: Basic and Preclinical Cancer Research

This section contains 18 subsections, which review pleiotropic MTF effects in specific contexts. 

### 3.1. Mitochondrial and Energetic Effects

MTF accumulates in the mitochondrial matrix in the presence of a polarized mitochondrial membrane potential and reversibly inhibits NADH dehydrogenase (mitochondrial complex I) of the respiratory chain by repressing efficient coupling of the redox and proton transfer domains resulting in suppression of ATP production [15,17,30,31,32]. One of the studies that examined the inhibitory effects of MTF on mitochondrial complex I in cancer cells used the MTF-resistant *Saccharomyces cerevisiae* NADH dehydrogenase (Ndi1) that overexpressed in cell culture and in xenograft tumors in mice had no inhibitory effects on cancer cell growth under MTF treatment compared to controls showing impaired proliferation [17]. Of note, MTF induced cell death under glucose deprivation in control cells but not in Ndi1 overexpressing cells. 

A biochemical study in cell culture systems indicated that the dose-dependent inhibition of MTF on cancer cell proliferation is probably exerted by a metabolic switch to glycolysis as a consequence of MTF-dependent suppression of mitochondrial complex I [33]. This limits the flux of glucose- and glutamine-derived intermediates into the tricarboxylic acid (TCA) cycle that in turn limits acetyl-CoA levels necessary for lipid biosynthesis. Of note, the metabolic switch was also detected in AMPK-deficient cells indicating that the antiproliferative effect of MTF does not depend on AMPK. Furthermore, MTF could not inhibit cell proliferation under hypoxia.

In rodents, a biochemical study demonstrated that the redox shuttle enzyme mitochondrial glycerol-3-phosphate dehydrogenase (GPDH) is another primary MTF target where the drug increases the cytosolic and decreases the mitochondrial redox state (in plasma and liver), reduces conversion of lactate and glycerol to glucose, and decreases hepatic gluconeogenesis [34]. These effects were achieved using MTF concentrations equivalent to those observed in diabetic patients. In thyroid cancer, compared to normal thyroid tissue, mitochondrial GPDH was found to be overexpressed [35]. Cell culture experiments indicated that thyroid cancer cells with high mitochondrial GPDH expression are more susceptible to MTF-induced inhibitory effects of OXPHOS. Growth inhibitory effects were induced by MTF in cell culture and in metastatic thyroid cancer in mice. 

Hexokinase II is attached to the outer mitochondrial membrane and highly expressed in cancer cells [36,37]. Cell culture assays and molecular modeling studies indicated that MTF impairs directly glucose metabolism by inhibiting hexokinase II activity through occupying the binding site for glucose-6-phosphate. This induces hexokinase II dissociation from mitochondria leading to activation of apoptotic signaling.

Cancer cells have a higher demand for ATP than non-transformed cells, which has vital consequences under MTF treatment. As a result, glucose concentrations in cancer are often three to 10 times lower than in unaffected tissues [38]. In low glucose media, cell lines either with mitochondrial DNA mutations affecting complex I subunits or with impaired glucose utilization were more sensitive to MTF or phenformin, an antidiabetic drug similar to MTF, compared to cell lines without these metabolic impairments. Therefore, glucose concentrations are a considerable factor when assessing sensitivity of cancer cells to OXPHOS inhibitors such as MTF. 

In breast cancer cells and non-transformed control cells, where MTF decreased mitochondrial respiration resulting in lower ATP generation and in increased aerobic glycolysis, the comparably larger increase in glycolysis by breast cancer cells could not compensate for the greater demand of these cells for ATP to sustain proliferation [39].

### 3.2. Gastrointestinal Tract Effects

One of the major effects of MTF in the liver is to reduce gluconeogenesis resulting in specific reduction of hepatic glucose output. Recent studies present evidence that the gastrointestinal tract constitutes another important site where MTF exerts its glucose lowering effects [23,40,41,42,43]. Increased glucose utilization and lactate production are associated with MTF and the intestine contributes to this effect considerably [23,42,44]. Of note, a study in mice demonstrated that intestinal glucose-1-^13^C uptake led to a partial liver production of glucose-1,6-^13^C. This process is presumably associated with net loss of ATP when summing-up glycolysis in the intestinal wall and gluconeogenesis in the liver [44]. In this regard, a metabolic study in volunteers and in rodents treated with MTF reported that increased plasma lactate and pyruvate concentrations were attributed to an increased anaerobic glycolysis which is known to generate less ATP than aerobic glycolysis [45]. 

### 3.3. AMPK-Dependent Mechanisms

AMPK is considered as one of the major regulators of the cellular energy state and of key cellular processes such as lipid and glucose metabolism, cell growth, autophagy, and apoptosis [46]. The enzyme maintains mitochondrial homeostasis and is activated when the AMP or ADP to ATP ratios increase. Energy loss is compensated by upregulating glycolysis. The upstream regulator liver kinase B1 (LKB1) directly activates AMPK by phosphorylation at thr172 and is required for MTF-induced AMPK activation [47]. Cell culture experiments, immunoprecipitation, and pull-down assays demonstrated that MTF activation of AMPK requires the interaction of the LKB1 with the inositol polyphosphate multikinase (IPMK) [48]. In addition, knockout experiments indicated that the lysosome pathway is necessary for MTF activation of AMPK [49]. 

MTF indirectly activates AMPK by phosphorylation that in turn downregulates expression of the critical lipogenic transcription factor sterol regulatory element binding transcription factor 1 (SREBF1; alias, SREBP-1), resulting in downregulation of lipogenic enzymes such as Fas cell surface death receptor (FAS) and ribosomal protein S14 (S14) [18].

In vitro studies in prostate and ovarian cancer cells indicated that the LKB1/AMPK axis is required for MTF-induced increased histone and non-histone acetylation via AMPK-mediated increased inhibitory phosphorylation of acetyl-CoA carboxylase (ACC) [50]. Inactivation of ACC through phosphorylation is also a mechanism to stimulate fatty acid oxidation. In hepatocytes, the effect of MTF, by indirectly activating AMPK, resulted in reduced ACC activity, induced fatty acid oxidation, and repressed expression of lipogenic enzymes [18]. Using the reversible AMPK inhibitor compound C in hepatocytes, the MTF-initiated inhibition of glucose generation was reversed. 

The mechanistic target of rapamycin (mTOR) complex 1 (mTORC1), which includes as essential components mTOR and the scaffolding protein regulatory associated protein of MTOR complex 1 (RPTOR), acts downstream of AMPK and represents another crucial regulator of metabolism. The lowest levels of MTF that inhibit mTORC1 require AMPK and the tuberous sclerosis complex protein complex (TSC complex) for signaling [51].

### 3.4. AMPK-Independent Mechanisms

In addition to AMPK-mediated MTF effects, several AMPK-independent mechanisms have been described, underscoring the pleiotropic effects of MTF in cancer [30,52]. An AMPK-independent mechanism of reducing glucose levels by MTF involves suppressing glucagon signaling. In vitro and in vivo experiments indicated that MTF suppresses glucagon-dependent glucose output from hepatocytes by a mechanism involving accumulation of AMP and related nucleotides, which suppresses adenylyl cyclase, reduces levels of cyclic AMP, and reduces protein kinases A activity [53,54]. Knockout of AMPKα1 and AMPKα2 catalytic subunits in mice demonstrated that MTF impedes hepatic gluconeogenesis by reducing the hepatic energy state independently of the LKB1/AMPK pathway [55]. 

An AMPK-independent mechanism of MTF effects was demonstrated in hepatoma cell lines where hypoxia inducible factor 1 subunit alpha (HIF1A) protein accumulation was impaired by MTF-mediated HIF1A protein degradation [56]. Decreased immunohistochemical expression of HIF1A in hepatoma xenografts in mice supported the findings. In addition, decreased expression was revealed for solute carrier family 2 member 1 (SLC2A1; alias, Glut1), and carbonic anhydrase 9 (CA9; alias, CAIX). 

In prostate cancer cells with an intact p53 gene, MTF was more effective in reducing proliferation than in prostate cancer cells with impaired or absent p53 activity [57]. This observation may have implications for the use of MTF in prostate cancer as a considerable number of cases harbor p53 mutations. Furthermore, the AMPK-independent antiproliferative effect of MTF in prostate cancer cells with intact p53 was mediated by MTF-induced upregulation of the DNA damage inducible transcript 4 (DDIT4; alias, REDD1). DDIT4 is a direct target of p53 and its induced expression was associated with inhibition of mTOR expression.

### 3.5. Micro RNAs

Micro RNAs (miRNAs) are implicated in tuning gene expression and are commonly deregulated in cancer. Cell culture experiments indicated that MTF exerts regulative functions on a number of miRNAs [58,59]. miRNAs that were reported to be upregulated upon MTF treatment included let-7 miRNAs, miR-26a, and miR-34a, whereas miR-181a, miR-221, and miR-222 were downregulated. In the case of miR-26a, MTF treatment and increased expression of this miRNA resulted in cell growth inhibition of renal cancer cells and in impaired cell viability of breast cancer cells [60,61]. The BCL2, apoptosis regulator (BCL2) was one of the downregulated genes during MTF treatment. 

### 3.6. Stress Effects

Limited energy supply and restricted vascularization renders tumor cells sensitive to stress. One of the central MTF effects on cancer cells is to enhance energy stress, especially under low glucose levels. This fact has further implications on stress-related mechanisms, as exemplified by the following surveys. 

By inhibiting mitochondrial complex I, MTF renders cells sensitive to glucose deprivation as the unfolded protein response (UPR) mediated by the ER, to reduce accumulation of unfolded proteins in the ER under stress conditions, requires a functional respiratory chain for activation [62]. 

A stress-related effect of MTF was studied in cancer cell cultures and in xenograft tumors in mice [63]. MTF treatment induced mitochondrial swelling due to ER stress as a result of calcium flux from the ER to the mitochondria. This process initiated antiapoptotic response mechanisms even though MTF exerted antitumorigenic effects in the xenograft tumors. Upregulated stress-related factors included, e.g., DDIT4 and DDIT3, the latter being a pro-apoptotic factor associated with ER stress-mediated apoptosis.

In vitro and in vivo experiments in cancer cells demonstrated that forkhead box O3 (FOXO3) is a critical transcription factor under metabolic stress that, under this condition, is translocated in a cleaved form into mitochondria to support mitochondrial metabolism; however, MTF treatment mediated proapoptotic effects on mitochondrial FOXO3 via AMPK [64]. 

MTF treatment of ovarian cancer cells enhanced activation of the mitochondrial deacetylase sirtuin 3 (SIRT3) and intensified MTF-initiated apoptosis, energy stress, and mitochondrial malfunction, and, furthermore, increased AMPK overexpression [65].

Reactive oxygen species (ROS) are known to be a source of oxidative stress. In the doxorubicin-resistant breast cancer cell line MCF7/ADR, MTF impaired in a time- and dose-dependent manner the mitochondrial membrane potential [66]. Under low glucose levels but not under hypoxia, ATP levels were decreased and ROS levels were increased after short-term incubation with MTF. In addition, this study demonstrated in a xenograft mice model that intratumoral injection of MTF impairs tumor growth in a concentration-dependent manner.

### 3.7. Antiproliferative Effects

One of the central molecular mechanisms of MTF is the growth inhibitory effect on cancer cells that, depending on the context, may have further cellular consequences. Various cell culture models were employed to dissect the molecular events associated with MTF-induced antiproliferative effects. 

In colorectal cancer cells, for instance, MTF (5 mM) treatment inhibited proliferation and migration, resulting in an increased percentage of cells in the G0/G1 compared to the G2 cycle phase [67]. Decreased expression was revealed for MYC (alias, c-Myc), phosphorylated RB transcriptional corepressor 1 (p-RB1), and cyclin D1 (CCND1), which is implicated in the G1/S transition. Furthermore, MTF treatment slightly inhibited mTOR and more pronounced ribosomal protein S6 (RPS6) and 4E-binding protein 1 (4EBP1) through reducing site-specific phosphorylation. However, this effect did not correlate with enhanced p-AMPK in all examined cell lines. Inhibition of the mTOR/S6/4EBP1 axis is known to impair protein synthesis. Drug removal in the colorectal cell lines rescued cell proliferation and reactivated phosphorylation of insulin like growth factor 1 receptor (IGF1R) and molecules of the mTOR pathway. In addition, in two colorectal cell lines, strong mitochondrial depolarization coincided with increased ROS production, which contributed to the antiproliferative MTF effects. MTF also inhibited expression of the stem cell genes leucine rich repeat containing G protein-coupled receptor 5 (LGR5) and CD44. The researchers discussed that absence of apoptosis in their experiments is presumably attributed to the fact that the cells were cultured under low-density conditions that lowered effects of MTF-induced cytotoxic acidosis.

MTF (10 mM) treatment of colon cancer LoVo cells impaired cell viability [68]. Metabolic profiling of the cells revealed deregulation of energy metabolism in a time-dependent manner as exemplified by increased glutamate levels (8 h, 24 h, and 48 h), whereas glutamine levels were initially increased (8 h) but in long-term cell culture (48 h) decreased. 

In a xenograft mouse model of head and neck squamous cell carcinoma, photoacoustic imaging after MTF treatment (5 days, 200 mg/kg, i.p. injection) revealed a significant increase in tumor oxygen saturation and hemoglobin concentration [69]. Furthermore, tumor volume and immunostaining for proliferation marker Ki67 decreased, whereas staining for vascularity marker, platelet and endothelial cell adhesion molecule 1 (PECAM1; alias, CD31) remained unchanged. 

In a dose- and time-dependent manner, MTF inhibited proliferation, migration and invasion of osteosarcoma cells and this effect was associated with decreased expression of phosphorylated AKT serine/threonine kinase 1 (p-Akt) and vimentin (VIM), and increased expression of PTEN and cadherin 1 (CDH1) [70]. 

An expression profiling study in estrogen receptor positive MCF-7 breast cancer cells demonstrated that the antiproliferative effect of MTF is mainly a result of translational suppression of mRNAs of cell cycle regulators and tumor promoters, such as cyclin E2 (CCNE2) and ornithine decarboxylase 1 (ODC1), that are regulated via the mTORC1/4EBP protein pathway [71]. MTF is known to suppress this pathway.

### 3.8. Inhibition of Epithelial-to-Mesenchymal Transition 

Epithelial-to-mesenchymal transition (EMT) is a key process for promoting cancer progression. The transforming growth factor beta 1(TGFB1; alias, TGF-β) is known to be an inducer of the EMT process. In breast cancer patients treated with MTF, TGFB1 serum levels exhibited a trend reduction [72]. In lung adenocarcinoma cells, MTF inhibited TGFB1-induced phosphorylation of SMAD family member 2/3 (Smad2/3) dose-dependently by using the LKB1/AMPK axis [73]. Furthermore, MTF suppressed expression of the TGFB1 target genes plasminogen activator inhibitor type 1 (PAI1; alias, SERPINE1), fibronectin (FN), connective tissue growth factor (CTGF), and interleukin 6 (IL6). As exemplified by these and other studies, some of which are presented in a different context (Section 3.15 and Section 3.18), MTF is, under certain conditions, capable of inhibiting the EMT process.

### 3.9. Antiangiogenic Effects

Application of angiogenesis inhibitors has emerged as a therapeutic intervention in cancer treatment. One of the studies that assessed the antiangiogenic capacity of MTF in the clinical context was performed on a small number of resected hepatocellular carcinomas from diabetic patients with metabolic syndrome [74]. In patients who received MTF in their medication, vascular endothelial growth factor receptor (VEGFR) expression was comparably attenuated in the tumors. Other downregulated molecules included p-mTOR, CCND1, and ribosomal protein S6 kinase B1 (RPS6KB1). 

Cell culture experiments under normoxic conditions in uterine leiomyoma cells demonstrated that MTF downregulates VEGF protein levels in a dose-dependent manner [75]. Under hypoxic-like conditions, MTF suppressed HIF1A protein synthesis by inhibiting mTORC1 activity.

In cervical cancer xenografts in nude mice, MTF treatment reduced tumor growth and angiogenesis and this effect was associated with decreased binding of the inhibitor miR-142-3p to the 3′ untranslated region (UTR) of the long non-coding RNA metastasis associated lung adenocarcinoma transcript 1 (MALAT1) which is known as a tumor promoter in a number of cancer types [76]. The accompanied decreased expression of the architectural transcription factor high mobility group AT-hook 2 (HMGA2) upon MTF treatment was proposed to be an effect of miR-142-3p binding to 3′ UTR sequences of HMGA2.

In xenograft breast tumors, MTF treatment of mice resulted in decreased levels of platelet and endothelial cell adhesion molecule 1 (PECAM1; alias, CD31) which was used as an endothelial cell marker to examine changes of vascular branches [77]. Decreased levels of nuclear HIF1A were also revealed in the tumors. 

### 3.10. Autophagy 

Autophagy can promote survival of cancer cells under stress conditions but may also precede apoptosis. AMPK is regarded as one of the key regulators of autophagy [46]. In Ishikawa endometrial cancer cells, MTF treatment upregulated cyclin dependent kinase inhibitor 1A (CDKN1A; alias, p21), resulting in comparably more cells arresting in the G1 and G2/M phases [78]. Increased autophagy was detected by conversion of autophagosome component LC3BI to LC3BII and decreased levels of autophagosome target p62. In addition, apoptosis was induced, especially at a higher MTF concentration (10 mM). Apoptosis induction was detected by caspase (CASP3/7, -8, and -9) activities while inhibition of autophagy regulator beclin1 (BECN1) by siRNA silencing led to reduced apoptosis in MTF-treated cells. 

Studies in esophageal squamous cell carcinoma cells demonstrated that autophagy can be protective in inhibiting apoptotic cell death [79]. Furthermore, MTF treatment inactivated, by decreased phosphorylation, signal transducer and activator of transcription 3 (STAT3) and decreased expression of its downstream target BCL2.

The tumor suppressor p53 is mutated in about 50% of human cancers. In p53-wildtype colon cancer xenografts, MTF treatment did not affect tumor growth but initiated autophagy, whereas in p53-deficient colon cancer xenografts, MTF selectively inhibited tumor growth and induced apoptosis. These contrary findings were attributed to specific cytotoxic MTF effects on p53-deficient cells under these conditions [80]. 

### 3.11. Apoptosis

One of the main anticancer effects of MTF is the capability to induce apoptosis under certain cellular stress conditions. Several studies have investigated the molecular effects associated with the apoptosis process. In MCF-7 breast cancer cells, MTF (10 mM) treatment resulted in G1 phase cell cycle arrest, continuously reduced cell viability, and induced apoptosis during a 72 h post-treatment period [81]. These effects were mediated by increased levels of p53, CDKN1A, BCL2 associated X, apoptosis regulator (BAX), BCL2 associated agonist of cell death (BAD), and reduced levels of Akt, CCND1, BCL2, and MDM2 proto-oncogene (MDM2).

In the colon carcinoma cell line HT29, MTF induced both autophagy and apoptosis. On the molecular level, MTF increased, in a time- and dose-dependent manner, protein expression of apoptotic peptidase activating factor 1 (APAF1), cleaved poly (ADP-ribose) polymerase 1 (PARP1), autophagosomal marker microtubule associated protein 1 light chain 3 (MAPLC3), and CASP3 while decreasing expression of nuclear factor, erythroid 2 like 2 (NFE2L2; alias, NRF2) and nuclear factor-kappa B (NFKB) [82]. 

Studies in pancreatic cancer cells demonstrated that MTF treatment inhibited proliferation and induced apoptosis. These effects were associated with reduced expression of transcription factors Sp1, Sp3, Sp4, and pro-oncogenic Sp-regulated genes (e.g., BCL2, mTOR, VEGF, and MYC), indicating that one of the underlying mechanisms of MTF as an anticancer agent involves targeting Sp factors [83,84]. 

Glucose concentrations play a role in the MTF-induced apoptosis process. In an integrated metabolomics approach in HepG2 liver cancer cells, high dose of MTF mediated, under high glucose concentration, hypoglycemic effects, reduced tumor cell proliferation, and mediated apoptosis by activating AMPK/mTOR pathway signaling [85]. In two thyroid cancer cell lines, MTF (5 mM) induced cell death and oncosis at low (5 mM) but not at high (20 mM) glucose concentration [86]. This effect was probably induced by low ATP levels, which is likely the mechanism leading to cell death in low glucose medium. In similar manner, MTF induced apoptotic cell death at low glucose concentrations in different breast cancer cell lines, whereas a glucose concentration at supra-physiological conditions led in first instance to cell cycle arrest [87]. 

The mechanism of induced cell death under normal glucose concentration is associated with MTF-mediated inhibition of MYC [88,89]. In contrast, under high glucose conditions, MYC expression is not inhibited by MTF, and MYC induces upregulation of pyruvate dehydrogenase kinase 1 (PDK1) which inhibits pyruvate dehydrogenase (PDH), leading to enhanced glycolytic flux and resistance to MTF-induced cytotoxic effects.

### 3.12. Immune-Mediated Antitumor Response

A number of studies have demonstrated that MTF is capable of modulating the immune response in cancer. For example, an immune-mediated antitumor response was demonstrated in wildtype mice, where orally administered MTF protected CD8+ tumor-infiltrating lymphocytes from apoptosis and enhanced their multifunctionality by producing cytokines and promoting rejection of highly immunogenic tumors [90]. 

In lung metastases of melanoma cells in mice, MTF treatment induced a local and systemic anticancer immune response and lung infiltration of specific T cell populations was associated with MTF-induced antimetastatic effects [91]. Of note, only marginally antimetastatic effects of MTF were revealed in immunodeficient mice compared to wildtype mice. 

Using malignant cancer models in mice, MTF led to an accumulation of M1-like macrophages that, in contrast to M2-like macrophages, reduced cancer growth and angiogenesis [92]. In addition, the antiangiogenic effect of MTF in mice was demonstrated with high MTF concentrations (300 mg/kg daily), that reduced VEGF and fibroblast growth factor 2 (FGF2) expression in xenograft tumors.

### 3.13. Epigenetic Features

The effects on histone modifications and DNA methylation have been associated with MTF treatment of cancer cells, presumably promoting its anticancer properties via altered gene regulations [58]. For example, a series of cell culture experiments assessed the effect of MTF on changing DNA methylation patterns [59]. Through AMPK activation, MTF upregulated miRNA let-7 resulting in degradation of long non-coding RNA H19. Degradation of H19 relieved inhibition of S-adenosylhomocysteine hydrolase (SAHH), which in turn facilitated DNA methyltransferase 3B (DNMT3B) activity to modulate methylation patterns. These MTF-induced epigenetic effects were also revealed in endometrial cancer specimens. 

In ovarian cancer under normoglycemic levels (5.5 mM), MTF was more sensitive than under hyperglycemic levels (25 mM) to modify epigenetic profiles via AMPK activation, as indicated by repressed expression of histone H3 lysine 27 trimethylation (H3K27me3) and of components of polycomb repressor complex 2 (PRC2) [93]. 

### 3.14. Hematological Malignancies

Studies investigating MTF effects in hematological malignancies are less common. In two multiple myeloma cell lines, MTF applied up to a maximum concentration of 20 mM induced antiproliferative effects, autophagy and G0/G1 cell cycle arrest but did not initiate apoptosis [94]. In addition, MTF increased p-AMPK and reduced p-mTOR expression (ser2448 and ser2481) which repressed both mTORC1 and mTORC2 signaling pathways, attenuating expression of transcriptional key proteins such as 4EBP1 and p-Akt (ser473). In multiple myeloma xenograft mice models, MTF treatment (250 mg/kg daily) substantially sustained the in vitro findings by reducing tumor size, increasing expression of p-AMPK, and decreasing expression of p-mTOR and Ki67. 

In four lymphoma cell lines, the IC_50_ for four drugs, as single agents or in combination with MTF (10 mM), were determined, revealing that the response was cell type-specific [95]. In subsets of the lymphoma cell lines, MTF had a profound additive effect on decreasing the IC_50_ in combination with the BCL2 inhibitor venetoclax and in combination with the CDK9 inhibitor BAY-1143572. In promyelocytic leukemia cells, the antihypertensive drug syrosingopine (5 mM) significantly enhanced the sensitivity of MTF to induce cell death by reducing the IC_50_ of MTF from approximately 30 mM to approximately 2 mM [96]**.**

### 3.15. Cancer Stem Cells

Cancer stem cells (CSCs) are considered as a tumor-initiating and promoting cell population and are associated with chemo- and radiotherapy resistance in cancer. In vitro, CSCs are capable of forming tumorspheres and in vivo they have the capacity to generate tumors from a small number of cells under immunodeficient conditions. CSCs express a set of shared and unique stem cell markers, including CD44 and CD133, that are characteristic for different tumor types [97]. Frequently used model systems for CSC studies are breast cancer and glioma cells.

A mouse xenograft model of transformed mammary epithelial cells revealed that i.p.-applied MTF targets the CD44 high and CD24 low expressing CSC population and when combined with the anthracycline compound doxorubicin, known to target non-cancer stem cells, synergistically inhibits tumor growth [98]. The authors concluded that the selective targeting capacity of both drugs provides a rationale for their application in breast cancer treatment. In a HER2-overexpressing breast cancer cell line, resistant to trastuzumab, the breast cancer-initiating CD44+/CD24-/low population was more sensitive to MTF treatment than the non-CD44+/CD24-/low population (estimated IC_50_ for MTF 1 ± 0.2 mM vs. 11 ± 2 mM) [99]. MTF in combination with trastuzumab significantly reduced xenograft tumor growth in mice, indicating that a combination of both drugs may gain clinical relevance. 

Cell death of CSCs derived from human mammary adenocarcinomas and characterized by a CD44 high/CD24 low expression profile was preferentially mediated by MTF in concentrations applied in a range used in T2D patients [100]. Metabolic profiling in CSCs derived from mammospheres revealed that MTF treatment leads to elevated levels of folate and homocysteine and, similar to phenformin, exhausts levels of nucleotide triphosphates (NTPs and dNTPs) that is likely a result of impaired OXPHOS [101]. 

Using stem-like glioma-initiating cells derived from different human glioblastoma multiforme (GBM) samples, MTF activated AMPK which, in turn, activated differentiation marker FOXO3 as a transcription factor [102]. This activation resulted in differentiation of the cells, as indicated by reduced expression of neural stem cell/progenitor markers nestin (NES), Musashi RNA binding protein 1 (MSI1), and BMI1 proto-oncogene, polycomb ring finger (BMI1) and elevated expression of differentiation markers glial fibrillary acidic protein (GFAP) and βIII-tubulin. Lowering the glucose concentration of the medium from 26.2 mM to 17.5 mM, MTF (1 mM) phosphorylated more efficiently both AMPK and the AMPK downstream target ACC. I.p. injection of MTF (500 mg/kg) did not significantly impair growth of xenograft tumors, established from implanted stem-like glioma-initiating cells. However, dissociated cells, reimplanted into nude mice, exhibited impaired secondary tumor growth, demonstrating that MTF treatment led to depletion of stem-like glioma-initiating cells. 

MTF (10 mM) applied to brain tumor initiating cell (BTIC) lines established from stage IV gliomas exerted inhibitory effects on migration and proliferation in a subset of the established cell lines [103]. In one MTF-sensitive BTIC line, proliferation was significantly inhibited at a low MTF concentration (0.01 mM thrice daily) which, according to the report, is likely to be achieved in human brain tumors. 

In two GBM cell lines, MTF was capable of suppressing expression of CSCs markers MSI1, BMI1, SRY-box 2 (SOX2), as well as zinc finger E-box binding homeobox 1 (ZEB1), the latter also known as a critical EMT-associated transcription factor [104]. Furthermore, MTF suppressed the TGFB1-initiated EMT-like process via downregulation of EMT-related factors cadherin 2 (CDH2; alias, N-cadherin), vimentin (VIM), and snail family transcriptional repressor 1 and 2, respectively (SNAI1 and SNAI2).

In the rat model of N-methyl-N-nitrosourea-induced bladder cancer, MTF was not capable of suppressing bladder cancer initiation but repressed progression from mild to moderate/severe dysplasia and from carcinoma in situ to invasive carcinoma [105]. MTF treatment increased the apoptotic cell population in the tumors and reduced the number of cells positive for stem cell markers keratin 14 (KRT14; alias, CK14) and POU class 5 homeobox 1 (POU5F1; alias, OCT3/4). Reduction of the CSC population was mediated at lower MTF concentrations (<10 mM) through decreased protein levels of mitochondrially encoded cytochrome c oxidase II (MT-CO2; alias, COX2), prostaglandin E (PGE2), and p-STAT3. 

These and other, herein not presented, CSC studies indicate that MTF has the capacity to specifically target CSCs that is of relevance for depleting tumors from those cells which confer chemoresistance and form the most proliferatively active and progressive tumor cell population. The diverse mechanisms of MTF actions on CSCs at the molecular level have been outlined in detail in a recent review [97]. MTF is capable of targeting CSCs through a number of signaling pathways including the Akt/PI3K/mTOR, insulin/IGF1, mitogen-activated protein kinase (MAPK), Sonic hedgehog (Shh), Wnt, TGFB, Notch, and NFKB pathways.

### 3.16. MTF in Combination with Other Anticancer Drugs 

Adding MTF to a chemotherapeutic regime is a strategy to enhance drug sensitivity. Several in vitro and in vivo studies have explored MTF with other compounds or drugs in various combinations, aiming to optimize and tailor therapy options. 2-deoxy-D-glucose (2DG) which is an AMPK activator and competitive inhibitor of glycolysis is frequently included in combination regimes. Gastrointestinal, brain, lung, liver, breast, ovarian, and pancreatic cancer models are commonly employed to assess combinatorial anticancer effects. 

In human gastric and esophageal cancer cell lines and in xenograft mouse models, MTF combined with 2DG sufficiently inhibited energy pathways, resulting in significant cell death accompanied with decreased cellular ATP, prolonged AMPK activation, and support of autophagy [106]. In colorectal cancer cells, oxaliplatin combined with MTF resulted in synergistic cytotoxicity, and limited expression of high mobility group box 1 (HMGB1), which is a regulator of cell death and survival [107]. In triple-negative breast cancer cells that were resistant to programmed cell death induced by extracellular matrix detachment (anoikis), the effect of combined treatment of MTF with 2DG was more pronounced to suppress proliferation and induce detachment of cells compared to single drug applications [108].

In a panel of non-small cell lung cancer (NSCLC) cell lines, MTF combined with an anti-IGF1R antibody inhibited IGF1R signaling and the combination of both MTF and anti-IGF1R antibody exerted additive inhibitory effects on cell viability [109]. In NSCLC cell lines that were resistant to EGFR tyrosine kinase inhibitors (TKIs), treatment with the pan-histone deacetylase inhibitor vorinostat and MTF synergistically improved sensitivity to the EGFR-TKI gefitinib [110]. Sensitivity was mediated by pro-apoptotic protein BCL2 like 11 (BCL2L11; alias, BIM) that induced apoptosis, whereas autophagy was significantly reduced by MTF. 

Gliomas are one of the most aggressive tumors and chemotherapy options are limited, partly due to exclusion of larger molecules to cross the blood–brain barrier. MTF, however, is able to cross the barrier. The synergistic effect of high MTF dose in combination with the alkylating agent temozolomide (TMZ) was demonstrated in a number of in vitro and in vivo studies in malignant gliomas. TMZ combined with MTF (both given at larger doses) showed the highest cytotoxicity after 48 h and 72 h treatment in GBM cells [111]. This kind of therapy regime was accompanied by the comparably highest cell apoptosis and efficiently increased p-AMPK but decreased expression of p-mTOR, p-Akt, and p53. A GBM xenograft mouse model that received i.p. injections of high dose MTF (10 mg/25 g daily) and TMZ (15 mg/kg daily) revealed the comparably highest survival time. Under these conditions, expression of fatty acid synthase (FASN), known to be associated with cancer aggressiveness, was strongly reduced in the xenograft tumors. Cell culture experiments employing two GBM cell lines revealed a synergistic inhibitory effect on cell proliferation of TMZ in combination with MTF in TMZ-resistant T98G cells, whereas only a slightly additive effect was demonstrated in TMZ-sensitive U251 cells [112]. Furthermore, MTF, as a single agent or in a combination treatment, increased the rate of glucose uptake and lactate release in both cell lines more pronounced than single TMZ application. In T98G xenograft tumors in mice, combination therapy inhibited tumor growth more efficiently than single drug applications. The authors concluded that MTF might benefit those patients who experience TMZ resistance.

The mechanisms of MTF effects in combination with several chemotherapeutic drugs acting as hormone modulators, anti-metabolites, antibiotics, or inhibitors of DNA function or protein synthesis have recently been reviewed in detail [113]. For example, combining MTF with anti-hormone chemotherapeutic drugs mediates synergistic effects by affecting gene transcription and hormone signaling pathways, resulting in cell cycle arrest and apoptosis. Treatment aims of MTF in combination with antibiotics are to reduce cardiotoxicity. The combination of MTF with drugs, such as cisplatin, that interfere with DNA functions, induces apoptosis and sensitizes cancer cells to the cytotoxic effects of the chemotherapeutic drug. The mechanisms of MTF with taxanes known to impair protein synthesis include downregulation of ERCC excision repair 1, endonuclease non-catalytic subunit (ERCC1) and inhibition of lipid and cholesterol synthesis. In addition to combinatorial applications with chemotherapeutic agents, the antitumorigenic capacity of MTF has been successfully assessed in combination with natural compounds such as quercetin (5,7-dihydroxyflavone), chrysin (5, 7-dihydroxyflavone), flavone (2-phenyl-4H-1-benzopyran-4-one), and with the pain reliever and anti-inflammatory drug aspirin [114,115,116,117]. 

These in vitro and in vivo studies demonstrate, in their majority, that MTF in combination with other drugs acts in an additive or synergistic manner to enhance anticancer effects. These findings are of importance since MTF is well tolerated and is practical to lower doses of cytotoxic chemotherapeutic agents in combination therapies without losing effectiveness. In this regard, a number of concurrent retrospective and randomized studies in NSCLC are assessing MTF in combination with standard treatments [118].

### 3.17. Drug Delivery

New drug delivering systems are examined to improve efficiency of MTF as a single agent or in combination therapy. For example, MTF and curcumin co-encapsulated in PEGylated poly(lactic-co-glycolic acid) nanoparticles exhibited a synergist antiproliferative effect on T47D breast cancer cells compared to single encapsulated or non-encapsulated agents [119]. Dual drug loaded nanoparticles were associated with higher cytotoxicity and apoptosis in doxorubicin-resistant breast cancer cells compared to the corresponding free-administered compounds [120]. 

An MTF-extended release formulation compared to the standard application formulation was examined in T2D patients for 24 weeks in a clinical trial (NCT01864174) [121]. Safety and efficacy profiles, e.g., decrease in HbA1c levels, were similar in both groups with the advantage of the once-daily application in the extended release formulation group. 

To increase local MTF concentrations at tumor sites, a thermosensitive gel formulation has been developed allowing significant MTF accumulation within tissues and a more continuous release of MTF at the application site [122]. In a xenograft mouse model, MTF peritumorally applied at 100 mg dosages with the gel formulation to pseudo-orthotopic human breast cancer cell xenografts, led to increased local MTF concentrations, resulting in significantly lower tumor growth with a significantly lower number of mitotic cells and peripheral vessels. In contrast, MTF applied as an aqueous solution seemingly did not impair tumor growth.

### 3.18. MTF-Associated Resistance and Gene Polymorphisms

Resistance or variations in response to MTF treatment have been observed in cancer model systems and in the clinical context. For example, limitations of MTF anticancer effects were demonstrated in non-obese, non-diabetic rodent cancer models including the hydroxybutyl(butyl)nitrosamine-induced Fischer-344 rat model of invasive urinary bladder cancer [123]. In this model, MTF treatment failed to decrease cancer incidence, even though MTF treatment resulted in p53 phosphorylation in tumors in two of three investigated serine residues. In addition, in a rat model of oral cancer and in a mouse model for multiple intestinal neoplasia, MTF was ineffective.

The underlying mechanisms of MTF-sensitive and MTF-resistant cells were studied in detail in cancer cell lines and in patients-derived cell culture lineages [124]. Expression profiling and gene sequencing identified genetic variations that, even in different cell populations derived from the same patient, are helpful to explain heterogeneous responses to MTF treatment and may gain relevance for optimizing MTF treatment schemes.

Resistance of renal cell carcinoma cells to long-term MTF treatment was associated with diminished histone H3 acetylation and upregulation of EMT and stem-like markers. Adding valproic acid, which is a known inhibitor of histone deacetylases, to the MTF-treated cells could override resistance, and this effect was associated with enhanced histone H3 acetylation and reversion of the EMT process [125]. Synergistic cytotoxicity of the combination treatment was demonstrated, e.g., by reduced levels of p-Akt, p-SMAD3, and CDH2.

A CSC-related MTF resistance was observed in a panel of colorectal cancer cells. MTF (10 mM) decreased the ratio of CD44+/CD133+ CSCs, in four colorectal cancer cell lines, whereas in four other colorectal cancer cell lines, MTF did not disturb the number of CSCs [126]. A dose-dependent reduction of the number of tumorspheres was observed in an MTF-sensitive cell line. In one MTF-resistant cell line, in vitro experiments demonstrated that MTF resistance is caused by a compensation effect where glucose is substituted by glutamine as an energy source. Consequently, combined treatment with MTF and a glutaminase C inhibitor, impaired the ratio of CSCs in the MTF-resistant cell line. These results were sustained in xenograft mice models. The influence of glucose concentrations on MTF anticancer effects was studied, e.g., in high-grade serous ovarian cancer cell lines, which were resistant to MTF [127]. Low glucose concentrations sensitized the cell lines to MTF treatment, resulting in cell proliferation inhibition**.**

The mechanisms of acquired MTF resistance have been investigated at the molecular level in the MCF-7 breast cancer cell line and its MTF-resistant MCF-7 derivate [128]. A constitutive increased expression of SNAI1, a key marker of EMT transition, was demonstrated in MTF-resistant cells, whereas a profound decrease of CDH1, a key epithelial marker of cancer cells, was revealed in the resistant cells. In addition, p-Akt expression was comparably upregulated in the resistant cells. 

Thus far, few studies have addressed gene polymorphisms that are associated with responses to MTF application. In human, OCT1 is the major hepatic uptake transporter for MTF, and certain non-synonymous polymorphisms in OCT1 impair its functionality, resulting, for example, in higher MTF maximal plasma concentrations [129]. A meta-analysis on genome-wide association studies (GWAS) for MTF response, comprising more than 13,000 individuals, found an association of an SNP in the solute carrier family 2 member 2 (SLC2A2) with glycemic response to MTF [130]. SLC2A2 encodes the GLUT2 glucose transporter. 

Genotyping in a case series comprising over 1300 T2D patients revealed that common and rare variants in pre-mRNA processing factor 31 (PRPF31), carboxypeptidase A6 (CPA6), and STAT3 are associated with MTF response in the patient group [131]. 

**Figure 1:**
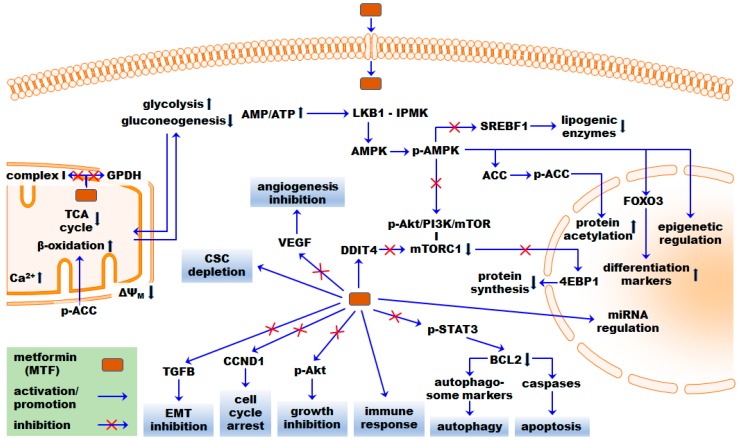
Putative anticancer effects of MTF exemplified by molecular and cellular key events. MTF affects key energy and metabolic processes such as the mitochondrial respiration (complex I), TCA cycle, fatty acid β-oxidation, gluconeogenesis, and glycolysis. MTF affects cellular fate processes such as cell cycle, cell growth, EMT, autophagy, and apoptosis. AMPK, the cellular key energy sensor, is phosphorylated in response to an increased AMP/ATP ratio and implicated in exerting several pleiotropic MTF effects. AMPK-dependent mechanisms include, e.g., inhibition of 4EBP1 via the Akt/PI3K/mTOR pathway. AMPK-independent mechanisms include, e.g., inhibition of mTORC1 by DDIT4. The pleiotropic effects of MTF on gene regulation include, e.g., downregulation of differentiation markers and modulation of epigenetic and mRNA features. Direct activation or inhibition processes are thoroughly known only for a limited number of molecules, such as LKB1 activation of AMPK. ΔΨ_M_, mitochondrial membrane potential; 4EBP1, 4E-binding protein 1; ACC, acetyl-CoA carboxylase; Akt, AKT serine/threonine kinase 1; AMPK, AMP-activated protein kinase; BCL2, apoptosis regulator, BCL2; CCND1, cyclin D1; CSC, cancer stem cell; DDIT4, DNA damage inducible transcript 4; EMT, epithelial-to-mesenchymal transition; FOXO3, forkhead box O3; GPDH, glycerol-3-phosphate dehydrogenase; IPMK, inositol polyphosphate multikinase; LKB1, liver kinase B1; miRNA, micro RNA; mTORC1, target of rapamycin complex 1; SREBF1, sterol regulatory element binding transcription factor 1; STAT3, signal transducer and activator of transcription 3; TCA, tricarboxylic acid; TGFB1, transforming growth factor beta 1; VEGF, vascular endothelial growth factor. Phosphorylated molecules are indicated by a prefix p.

Taken together, several studies presented in Section 3 demonstrate that MTF, by targeting in first instance the mitochondrial respiration, exerts its pleiotropic anticancer effects on key metabolic, energy and cellular processes including those affecting angiogenesis, proliferation, autophagy, apoptosis, and EMT (Figure 1). Key molecules include, e.g., ACC, AMPK, BCL2, 4EBP1, STAT3, TGFB, and the Akt/PI3K/mTOR pathway genes [46,132,133]. More research studies would be practical to assess the relation between molecular and metabolic MTF effects directly in tumors of rodent models.

## 4. MTF Effects on Cancer: Population-Based Studies, Clinical Studies and Trials, and Recent Meta-Analyses

### 4.1. Population-Based Studies

Retrospective cohort studies aim to collect information on effectiveness, side effects, safety, interactions, and dosage to improve design of clinical trials. Several observational studies have estimated the general cancer risk and the risk for specific cancer sites comparing MTF users with non-MTF users, especially in diabetic patients.

In 2005, a Scottish observational pilot study on registered data of more than 300,000 residents of Tayside reported for the period 1993–2001 that fewer newly diagnosed cancer patients who were admitted to hospitals were identified in T2D MTF users compared to T2D non-MTF users (adjusted odds ratio (OR) 0.77, 95% confidence interval (CI) 0.64–0.92) [134]. For the observational period 1994–2003, the same research group reported later that 7.3% of 4085 T2D MTF users were diagnosed with cancer compared to 11.6% of 4085 matched T2D non-MTF users (adjusted hazard ratio (HR) 0.63, 95% CI 0.53–0.75) [135]. A relation was also found between lower cancer incidence in the group taking the highest MTF dose over a longer period. Significantly reduced cancer risk was identified for bowel cancer.

A retrospective cohort study including nearly 63,000 diabetic patients admitted to general practices in the UK, found that MTF as single agent reduced cancer risk, especially for colorectal and pancreatic cancer when compared to sulfonylurea, MTF plus sulfonylurea, or insulin-based treatment [136]. In the Clinical Practice Research Datalink, a UK-based retrospective cohort study that included over 51,000 MTF users with T2D and over 18,000 MTF users with T2D who started to use sulfonylurea, risk for any type of cancer was similar in both groups when recorded with a time lag of one year after drug treatment initiation [137].

In the Zwolle Outpatient Diabetes project Integrating Available Care (ZODIAC) study with a mean follow-up of 9.6 years for 1353 registered participants, the adjusted HR for cancer mortality was 0.43 (95% CI 0.23–0.80) for T2D patients compared to non-MTF users [138]. Dose–response analysis found a decrease of mortality with increasing MTF dosage. Sulfonylurea use and macrovascular complications as confounders or exclusion of the first three years after enrollment did not significantly influence cancer mortality.

The Korean National Diabetes Program (KNDP) reported, after a mean follow-up of 5.8 years and adjusting for demographic and clinical parameters and excluding patients diagnosed with cancer within the first year after enrollment, a reduced cancer risk for MTF users (HR 0.513, 95% CI 0.318–0.826, *p* = 0.006) [139]. In a Taiwanese population-based cohort study, on nearly 801,000 individuals registered with the National Health Insurance system and cancer-free in the first year of study enrollment, MTF (≤500 mg daily) in T2D users significantly reduced total cancer risk to a similar level as seen for the non-T2D population [140]. The benefit of MTF application for individual tumor sites varied, partly to the extent to which possible confounding factors were considered. A significant benefit of MTF use to lower risk was revealed for colorectal (adjusted HR 0.36, 95% CI 0.13–0.98), liver (adjusted HR 0.06, 95% CI 0.02–0.16), and pancreatic cancer (adjusted HR 0.15, 95% CI 0.03–0.79); however, the benefit of MTF use was gender-specific for colorectal and liver cancer. Another Taiwanese population-based study on colorectal cancer reported that risk reduction for this entity in T2D patients was associated with MTF treatment in a dose-dependent manner [141].

A cohort study reported that MTF application for at least five years in T2D patients registered with a care consortium (USA location) resulted in risk reduction for colorectal cancer in the entire membership (HR 0.78, 95% CI 0.60–1.02), in current MTF users (HR 0.78, 95% CI 0.59–1.04), and in men (HR 0.65, 95% CI 0.45–0.94) [142].

In diabetic men with newly diagnosed prostate cancer, a cumulative duration effect for each additional six months of MTF treatment was seen in a Canadian retrospective cohort study in prostate cancer-specific death (adjusted HR 0.76, 95% CI 0.64–0.89, *p* = 0.001) [143]. However, the association of MTF with decreased all-cause mortality declined over time. 

A large population-based prospective cohort study (Prostate Cancer data Base Sweden, PCBaSe) on prostate cancer in T2D patients reported a decreased cancer risk only for insulin and sulfonylurea users but not for MTF users (HR 0.96, 95% CI 0.77–1.19) [144]. These findings may support a presumably protective role of T2D for prostate cancer [145].

Taken together, several population-based studies including the aforementioned studies have reported that MTF is reducing cancer incidence in diabetic patients, especially for some major cancer sites including colorectal, liver, and pancreatic cancer, whereas, for some other tumor sites, MTF obviously has minor or no significant positive association [3]. Of note, MTF treatment is seemingly associated with a cumulative dose and duration effect. Critical reviews on epidemiological and observational studies on MTF application in cancer have stated that these studies vary in design, and the majority are at certain risk of bias affecting different domains such as time-related confounders, exposure definition, and baseline adjustments [146,147]. Therefore, the outcome of these studies must be seen in the context that confounding factors are, in general, less controlled than in clinical trials. Even though observational cohort studies have limited ability to control certain confounding factors, improved experience to design and perform such studies has apparently resulted in an increase of the methodological quality scores in recent years, based on the Newcastle–Ottawa Scale [148].

### 4.2. Clinical Trials and Studies

Clinical trials involving MTF as a therapy option are focusing predominately on the most frequent cancer types. About one-third of clinical trials listed in June 2018 in the EU Clinical Trials Register involve MTF treatment of breast cancer patients (https://www.clinicaltrialsregister.eu). Other abundant trails include, e.g., gynecological and prostate cancer. In a number of National Health Institute (NHI) approved clinical phase 1 and 2 trials, MTF is assessed as single agent or in combinations with other repurposed drugs, i.e., disulfiram and chloroquine, for treatment of GBM [149]. A recent study summarized that nearly 70% of clinical trials involving MTF are applying the drug in combination with cytotoxic chemotherapy [150]. For example, in a phase III trial, MTF was successfully applied in combination with lapatinib and/or trastuzumab to improve distant disease-free and overall survival in diabetic patients with HER2-positive, early breast cancer [151]. The following selection of studies and trials comprises those which are commonly not included in meta-analyses on MTF use in cancer.

A retrospective clinical study performed at a US cancer center on breast cancer patients who had received neoadjuvant therapy, reported that MTF treatment in diabetic patients was significantly associated with pathologic complete response when compared to diabetic non-MTF users [152]. Pathologic complete response was determined as no indication for invasive breast carcinoma and axillary lymph node metastasis at time of surgery.

In a small case series of patients who were followed-up after heart transplantation, only 4% of the T2D patients developed cancer in contrast to 62% of T2D patients who were not treated with MTF and 27% who were not T2D patients [153]. Two out of five diagnosed cancers were hematological malignancies in the T2D patient group receiving MTF, whereas skin cancer was most prevalent in the two other groups. 

In a phase I dose escalation trial on heavily pretreated cancer patients, the highest applied doses of the mTOR inhibitor temsirolimus (25 mg weekly) and MTF (2 g daily) were mostly tolerated by the majority of patients [154]. The treatment was accompanied by moderate promising effectiveness. 

For diabetic patients with pancreatic neuroendocrine tumors and receiving MTF, a retrospective clinical study reported that these patients had a significantly longer progression-free survival compared to patients without MTF treatment [155]. 

In a retrospective clinical study with limited numbers of participants, the effect of MTF application on complete response was assessed in MTF-treated and non-MTF-treated diabetic patients with thyroid cancer as well as in a non-diabetic control group [156]. Tumor size was significantly smaller in MTF-treated diabetic patients and risk of shorter progression-free survival was associated with diabetic non-MTF users. 

Several clinical studies have assessed MTF-related metabolic parameters and/or tumor biomarkers such as proliferation marker Ki67. In a four-week pre-surgery period, MTF treatment of non-diabetic breast cancer patients revealed a trend towards decreased Ki67 tumor expression in women with an elevated homeostasis model assessment (HOMA) index, whereas an opposite trend was revealed in women with a lower HOMA index [157]. In a neoadjuvant trial on a limited number of breast cancer patients, MTF (0.5 mg thrice daily) given as a neoadjuvant therapy resulted in a significant decrease of HOMA [158]. In addition, in a subset of cancer cases the Ki67 staining index significantly decreased whereas the Tunel apoptosis score increased. A clinical trial conducted by the NCIC Clinical Trials Group (NCIC CTG) MA.32 on non-diabetic patients with completed breast cancer surgery, reported that within six months, weight and a number of blood parameters including insulin, glucose, leptin, and C-reactive protein were significantly improved in the MTF group [159].

In a small pre-surgery trial on obese endometrial cancer patients, responders to MTF treatment were determined as those who had reduced Ki67 staining [160]. Concentration of a number of serum lipid metabolites changed in responders and expression increased for p-AMPK in tumors while it decreased for p-Akt, p-RPS6, and phosphorylated eukaryotic translation initiation factor 4E binding protein 1 (p-EIF4EBP1). Comparable results were obtained in a preoperative study on endometrial cancer where p-AMPK levels increased whereas levels of Ki67, p-RPS6, and p-ERK1/2 decreased under MTF treatment [161]. A study on atypical endometrial hyperplasia and endometrial cancer implemented an MTF non-user control group and the effect of MTF application during the pre-surgery period was detected in reduced Ki67 expression while other indicators displayed insignificant changes [162]. When using a threshold of 20% lowered immunohistochemical expression, one study on endometrial cancer did not detect reduced Ki67 expression during the pre-surgery period [163]. However, significantly reduced expression was revealed for p-Akt, and p-ERK1/2 (alias, p-p44/42MAPK). Although the study duration during the pre-surgery period is limited, these studies may be practical to identify those patient groups, which would benefit from prolonged MTF application. 

### 4.3. Recent Meta-Analyses 

Meta-analyses on MTF applications, especially in diabetic cancer patients, are frequently performed to summarize findings from studies of various designs and are practical to identify causative heterogeneity between study design and findings. They are also adequate to score the methodological quality of selected publications. However, inclusion and exclusion criteria for selecting studies for meta-analyses vary considerably. 

A meta-analysis on MTF as an adjuvant anticancer treatment reported in 2016 that diabetic patients with early stage prostate cancer and treated with radical radiotherapy benefited in terms of recurrence-free survival from MTF application (HR 0.45, 95% CI 0.29–0.70). No positive association of MTF use could be established with breast and urothelial cancer [164]. A meta-analysis, that was performed on diabetic cancer patients and overlapped in its content with the aforementioned meta-analysis, reported, in 2017, a significant reduction in all-cause mortalities for colorectal, endometrial, breast, prostate, and ovarian cancer, whereas, for liver, pancreatic, and lung cancer, no positive association could be established [165]. In addition, breast cancer was also significantly associated with reduced cancer-specific mortality.

A number of recent meta-analyses on specific cancer sites, reporting on the effect of MTF on cancer incidence and outcome, are presented in Table 1. Meta-analyses reporting recurrently on essentially the same datasets are not included. MTF application in gastric cancer patients significantly reduced the risk in a meta-analysis of all seven included studies (HR 0.763, 95% CI 0.642–0.905) but significance was lost if only non-Taiwanese studies were considered [166]. 

A meta-analysis on seven studies in colorectal adenomas reported no reduced risk for T2D MTF users compared to T2D non-MTF users (OR 0.86, 95% CI 0.66–1.12, *p* = 0.274); however, meta-analysis on three studies on advanced colorectal adenomas found a significant benefit of MTF usage in T2D patients (OR 0.51, 95% CI 0.41–0.63, *p* < 0.001) [167]. 

A meta-analysis on eight retrospective cohort studies including over 6000 T2D colorectal cancer patients who received MTF compared to nearly 5000 non-MTF receivers, found a significant improvement of overall survival (HR 0.82, 95% CI 0.77–0.87, *p* = 0.000); however, a subgroup analysis revealed a non-significant improvement of colorectal cancer-related survival (HR 0.84, 95% CI 0.69–1.02, *p* = 0.079) [168]. 

In a meta-analysis on 19 studies including more than 550,000 diabetic patients, MTF compared to non-MTF application decreased the ratio of liver cancer by 48% (OR 0.52, 95% CI 0.40-0.68, *p* < 0.001) [169]. Adjustment for a number of confounding factors did not significantly change the results; however, pooled analysis of the included two randomized clinical trials revealed no significant differences between MTF and non-MTF application. 

In two case-control and 13 cohort studies on lung cancer, a 23% reduced risk of all-cause mortality was revealed in diabetic MTF users compared to diabetic non-MTF users (HR 0.77, 95% CI 0.68–0.86, *p* < 0.0001) [170]. A decreased risk of progression or recurrence of lung cancer, reported in five cohort studies, was significantly associated with diabetic MTF users vs. diabetic non-MTF users (HR 0.50, 95% CI 0.39–0.64, *p* < 0.0001). 

A systematic review on the incidence of breast cancer and all-cause mortality in T2D patients assessed the association of MTF on breast cancer incidence in 12 studies and on mortality in 11 studies [171]. Although no association was observed between breast cancer incidence and MTF application (OR 0.93, 95% CI 0.85–1.03), a 45% risk reduction was detected for all-cause mortality (HR 0.55, 95% CI 0.44–0.70). However, the review also indicated a possible publication bias limiting the conclusions drawn from the observational studies. 

In a meta-analysis assessing the benefit of MTF use for endometrial cancer patients with diabetes, subset analysis of one case-control, one nested case-control, and three retrospective cohort studies revealed that MTF use was associated with a reduced relative risk for endometrial cancer incidence (relative risk (RR) 0.87, 95% CI 0.80–0.95, *p* = 0.006) [172]. However, the association was lost when only the case-control and nested case-control studies were examined. Another meta-analysis on endometrial cancer found a benefit for MTF use for overall survival in nine assessed studies (HR 0.58; 95% CI 0.45–0.76) and for progression-free survival in five assessed studies (HR 0.61; 95% CI 0.49–0.76) [173].

A meta-analysis on eight retrospective studies, including population-based as well as clinical-based studies, reported an improved overall survival in prostate cancer patients for MTF users vs. non-MTF users (HR 0.79, 95% CI 0.63–0.98) [148]. Pooled data for cancer-specific survival were determined from six studies (HR 0.76, 95% CI 0.57–1.02) and for recurrence-free survival from five studies (HR 0.74, 95% CI 0.58–0.95) indicating only significantly improved recurrence-free survival for MTF users. 

In a pooled analysis of nine retrospective cohort studies and two randomized controlled trials in diabetic pancreatic cancer patients, MTF use was significantly associated with improved survival (HR 0.86, 95% CI 0.76–0.97, *p* = 0.01) compared to non-MTF use [174]. In subgroup analyses, patients with resectable or locally advanced tumors, but not metastatic patients, benefited from MTF application. 

Together, these recent meta-analyses collaborate in part with a review reporting that MTF has beneficial effects, to a varying extent, on certain tumor sites [3]. Reported trend associations may imply a larger degree of heterogeneous responses among patients where biomarker assays may help to identify those patients who would benefit from MTF treatment. 

## 5. Summary

Several in vitro and in vivo studies indicate that MTF exerts its pleiotropic anticancer effects via key cellular and metabolic processes and molecular pathways. In addition, several studies have demonstrated that MTF specifically targets CSCs and exerts additive or synergistic effects in numerous combination treatments.

A mechanism that is likely to contribute to the limited efficacy of MTF observed in several studies and trials on diabetic patients is the applicable MTF concentration that is below the concentrations used in many in vitro and in vivo studies. However, the anticancer effects of MTF depend, in part, on the affected tumor site. Besides this, beneficial MTF effects have been observed in cancer-associated biomarkers and MTF has been repeatedly reported to be associated with reduced overall mortality of diabetic cancer patients, which might be, in part, attributed to beneficial MTF effects in reducing hyperglycemia and promoting weight loss and vascular protection [171,175,176]. It should be noted that MTF has enhanced anticancer effects under lower glucose levels, which can be a considerable factor in diabetic patients who are in the initial phase of MTF treatment. 

Further studies are desired that optimize formulas where MTF exhibits additive or synergistic effects with other anticancer compounds, especially in view of the fact that contraindications for MTF are limited. Considering the heterogeneous MTF responses in cancer patients, it can be envisaged that new test assays are developed to assess individual MTF responses. In addition, more quality observational studies and clinical trials with controlled confounding factors are desired that may lead to the enlargement of therapeutic indications of MTF. 

## Figures and Tables

**Table 1:** Meta-analyses of MTF effects on cancer-site-specific incidence and outcome.

Tumor Site	Study Design	Cancer Incidence	CSS/DFS/PFS/RFS	OS/ACM	Number of Participants	Reference
Gastric cancer	7 cohort	HR 0.763, 95% CI 0.642–0.905			>100,000	[166]
Colorectal adenoma	4 retrospective 1 retrospective cross-sectional 2 retrospective case-control	OR 0.86, 95% CI 0.66–1.12, *p* = 0.274			10,000–100,000	[167]
Advanced colorectal adenoma	3 retrospective	OR 0.51, 95% CI 0.41–0.63, *p* < 0.001			<10,000	[167]
Colorectal cancer	8 retrospective cohort (OS) 3 retrospective cohort (CSS)		HR 0.84, 95% CI 0.69–1.02, *p* = 0.079 (CSS)	HR 0.82, 95% CI 0.77–0.87, *p* = 0.000 (OS)	10,000–100,000	[168]
Liver cancer	10 cohort 9 case-control 2 RCTs	OR 0.52, 95% CI 0.40–0.68, *p* < 0.001			>100,000	[169]
Lung cancer	13 cohort, 2 case-control (OS) 5 cohort (DFS)		HR 0.50, 95% CI 0.39–0.64, *p* < 0.0001 (DFS)	HR 0.77, 95% CI 0.68–0.86, *p* < 0.0001 (OS)	10,000–100,000	[170]
Breast cancer	10 retrospective cohort, 1 prospective cohort, 1 nested case-control (cancer incidence) 11 retrospective (ACM)	OR 0.93, 95% CI 0.85–1.03		HR 0.55, 95% CI 0.44–0.70 (ACM)	10,000–100,000	[171]
Endometrial cancer	3 retrospective cohort 1 case-control 1 nested case-control	RR 0.87, 95% CI 0.80–0.95, *p* = 0.006			>100,000	[172]
Endometrial cancer	9 observational (OS) 5 observational (PFS)		HR 0.61; 95% CI 0.49–0.76 (PFS)	HR 0.58; 95% CI 0.45–0.76 (OS)	<10,000	[173]
Prostate cancer	7 retrospective cohort, 1 prospective cohort (OS) 6 retrospective cohort (CSS) 5 retrospective cohort (RFS)		HR 0.76, 95% CI 0.57–1.02 (CSS) HR 0.74, 95% CI 0.58–0.95 (RFS)	HR 0.79, 95% CI 0.63–0.98 (OS)	10,000–100,000	[148]
Pancreatic cancer	9 retrospective cohort 2 RCTs			HR 0.86, 95% CI 0.76–0.97, *p* = 0.01 (OS)	<10,000	[174]

ACM, all-cause mortality; CI, confidence interval; CSS, cancer-specific survival; DFS, disease-free survival; HR, hazard ratio; OR, odds ratio; OS, overall survival; PFS, progression-free survival; RCT, randomized controlled trial; RFS, recurrence-free survival; RR, relative risk.

## Abbreviations

2DG	2-deoxy-d-glucose
ACC	acetyl-CoA carboxylase
AMPK	AMP-activated protein kinase
CI	confidence interval
CSC	cancer stem cell
EMT	epithelial-to-mesenchymal transition
GPDH	glycerol-3-phosphate dehydrogenase
HOMA	homeostasis model assessment
HR	hazard ratio
IC_50_	half maximal inhibitory concentration(s)
i.p.	intraperitoneal
MTF	metformin
OR	odds ratio
OXPHOS	oxidative phosphorylation
T2D	type 2 diabetes
TCA	tricarboxylic acid
TMZ	temozolomide
